# Dioxin-Induced PAI-1 Expression: A Novel Pathway to Pancreatic β-Cell Failure in Type 2 Diabetes

**DOI:** 10.3390/ijms252211974

**Published:** 2024-11-07

**Authors:** Suyeol Im, Sora Kang, Woo Jung Son, Minuk Son, Seung Jun Oh, Hye Ji Yoon, Youngmi Kim Pak

**Affiliations:** 1 Department of Biomedical Sciences, Graduate School, Kyung Hee University, Seoul 02447, Republic of Korea; suryeol@khu.ac.kr (S.I.); ksr8947@khu.ac.kr (S.K.); dnwjd920@naver.com (W.J.S.); ohsungjun124@naver.com (S.J.O.); 2 Department of Physiology, School of Medicine, Biomedical Science Institute, Kyung Hee University, Seoul 02447, Republic of Korea; 3 Department of Neuroscience, Graduate School, Kyung Hee University, Seoul 02447, Republic of Korea; manjoo1062@gmail.com (M.S.); yhj2271@khu.ac.kr (H.J.Y.)

**Keywords:** diabetes, AhR, TCDD, PAI-1, β-cell failure

## Abstract

Exposure to environment-polluting chemicals (EPCs), which are ligands of the aryl hydrocarbon receptor (AhR), is associated with the development of type 2 diabetes (T2D). This study explores the mechanisms by which AhR ligands contribute to β-cell failure in T2D. Incubation of RINm5F rat pancreatic β-cells with low-dose 2,3,7,8-tetrachlorodibenzodioxin (TCDD), the most potent AhR ligand, inhibited glucose-stimulated insulin secretion (GSIS). A single injection of TCDD in wild type mice reduced the size of Langerhans islets, but not in AhR liver knock-out mice (AhR-LKO). RNA-seq database analysis identified *Serpine1*, encoding for plasminogen activator inhibitor type-1 (PAI-1) as a TCDD-mediated secretory protein that is synthesized in an AhR-dependent manner in the liver. Elevated PAI-1 levels were shown to induce Caspase-3/7-dependent apoptosis in RINm5F cells, suggesting a novel pathway through which EPCs exacerbate T2D. These findings support the hypothesis that chronic exposure to AhR ligands may directly inhibit GSIS in pancreatic β-cells and indirectly induce β-cell apoptosis through increased PAI-1. This study provides new insights into the EPC-PAI-1 axis as a missing link between pancreatic β-cell failure and the progression of T2D and offers a potential target for therapeutic intervention.

## 1. Introduction

By 2045, an estimated 629 million people worldwide are expected to have diabetes, a condition that the World Health Organization predicts will become the leading cause of death by 2030 [1]. This health crisis is primarily driven by type 2 diabetes (T2D), which accounts for 90–95% of all diabetes cases. While genetic predisposition, obesity, diet, and physical inactivity are recognized as risk factors for T2D, these factors alone do not fully explain the rapid increase in diabetes prevalence [1,2], suggesting the involvement of other contributing elements.

Emerging evidence has identified environment-polluting chemicals (EPCs) as significant contributors to the diabetes pandemic [3,4]. EPCs, including endocrine-disrupting chemicals (EDCs) and persistent organic pollutants (POPs), enter the human body through air, water, soil, and direct contact. These absorbed chemicals can promote diabetes, obesity, and other metabolic disorders by interfering with various physiological processes. However, while many EPCs have been shown to be epidemiologically correlated with the development of T2D and insulin resistance, there is no detailed mechanistic information on how these pollutants disrupt insulin metabolism. 

EPCs disturb metabolic homeostasis through mechanisms such as aryl hydrocarbon receptor (AhR) activation and mitochondrial dysfunction, both of which have been implicated in insulin resistance, T2D, and metabolic syndrome in numerous studies [5]. The AhR is a ligand-activated transcription factor that mediates the toxic effects of a wide range of EPCs, including dioxins. Among these, 2,3,7,8-tetrachlorodibenzodioxin (TCDD) is one of the most potent AhR ligands and has been extensively studied for its toxicological effects. Elevated serum concentrations of cocktails of AhR ligands (AhRL) and mitochondria-inhibiting substances (MIS) have been observed in diabetic patients, correlating with higher EPC burdens and metabolic disturbances [6]. Despite these associations, the precise AhRL concentration threshold and duration of exposure required to trigger T2D remain unclear.

A critical aspect of T2D development is the progressive decline in insulin sensitivity and β-cell function, as indicated by the homeostatic model assessment for insulin resistance (HOMA-IR) and HOMA-β indices, respectively. The significant decline in HOMA-β in patients progressing to T2D observed in a Korean community-based prospective cohort study (KoGES) emphasizes the critical role of β-cell impairment in the deterioration of glucose tolerance [7]. Although data are limited, pancreatic β-cells are clearly at risk of being targets of EPCs [8,9]. Low-dose POPs decreased insulin secretion by INS-1E β-cells [10]. EPCs have been linked to the dysfunction of β-cells [11] and insulin-responsive tissues [12] through various mechanisms, including oxidative stress, endoplasmic reticulum (ER) stress, mitochondrial dysfunction, impaired insulin exocytosis, apoptosis, inflammation, and altered calcium flux [13]. However, the specific factors and pathways involved in TCDD-induced β-cell impairment are not fully understood.

This study aimed to investigate the potential link between low-dose exposure to EPCs, specifically TCDD, and the development of T2D. In a mouse model designed to mimic systemic exposure to subtoxic levels of TCDD, we observed significant destruction of pancreatic islets following a single intraperitoneal injection (*i.p.*) in wild type mice, an effect not seen in liver AhR-deficient mice. To identify the factor(s) responsible for β-cell impairment, we conducted a database analysis of the liver transcriptomes of TCDD-exposed mice. Among the genes for secreted proteins with increased expression, serpin family E member 1 (*Serpine1*), which encodes plasminogen activator inhibitor type-1 (PAI-1), was notably elevated. PAI-1, a key regulator of the fibrinolytic system, is closely associated with metabolic disorders. PAI-1 is commonly found at elevated levels in T2D patients, primarily driven by obesity and insulin resistance rather than hyperglycemia [14,15,16]. This study employed both animal and cellular models to explore the hypothesis that PAI-1, upregulated by TCDD exposure, plays a causative role in β-cell failure, potentially offering novel insights into the pathogenesis of T2D.

## 2. Results

### 2.1. Low-Dose TCDD Inhibited Glucose-Stimulated Insulin Secretion (GSIS) in RIN-m5F Cells

**Figure 1:**
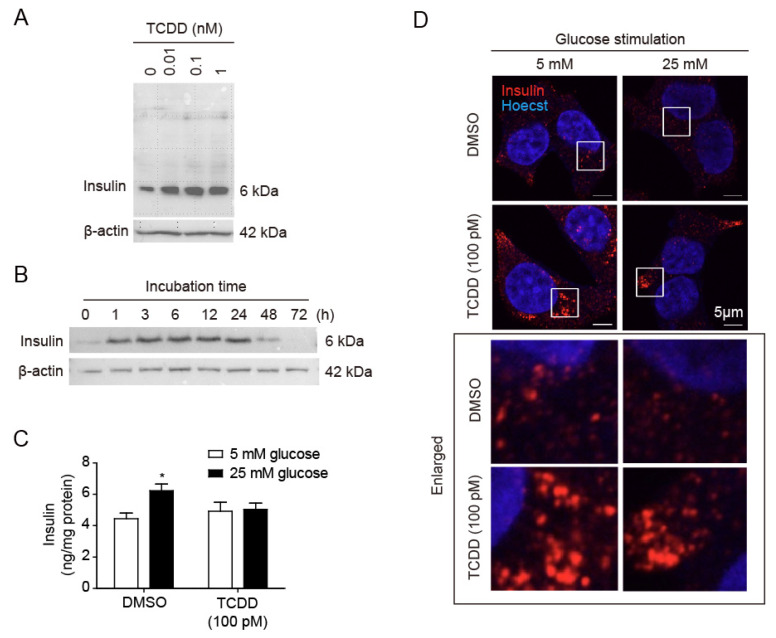
Effects of TCDD on insulin secretion in RIN-m5F pancreatic β-cells. Cells were treated with concentrations of TCDD and different periods of time, and the insulin levels were analyzed by different methods. (**A**) Western blot of cell lysates incubated with different concentrations of TCDD for 24 h, using β-actin as a loading control. (**B**) Western blot analysis of cell lysates after treatment with 100 pM TCDD for the indicated time periods. (**C**,**D**) Glucose-stimulated insulin secretion (GSIS). Cells incubated with or without 100 pM TCDD for 24 h were stimulated with 5 mM or 25 mM glucose for 1 h, culture media harvested for ELISA, and cells used for immunocytochemistry. (**C**) Quantification of secreted insulin by ELISA. Significant decrease in GSIS with TCDD treatment as compared to the control. Data represent the mean ± SEM (*n* = 3). * *p* < 0.05 vs. DMSO control. (**D**) Immunocytochemistry and confocal microscopy to visualize intracellular insulin. Enlarged white boxes from upper panels in lower panels.

To investigate the effect of TCDD on insulin secretion from pancreatic β-cells, RIN-m5F cells were incubated with TCDD in a time- or dose-dependent manner. Western blots of the cell lysates treated with 0, 0.01, 0.1, or 1 nM TCDD for 24 h showed a dose-dependent increase in intracellular insulin (Figure 1A). The amount of insulin in cell lysates increased up to 24 h after treatment with 100 pM TCDD (Figure 1B). To determine whether low-dose TCDD affects glucose-stimulated insulin secretion (GSIS), RIN-m5F cells were treated with 100 pM of TCDD for 24 h. Following treatment, ELISA, Western blots, and immunocytochemistry were performed. The TCDD inhibited GSIS, the insulin secretion stimulated by 25 mM glucose (Figure 1C). In addition, immunostaining with an insulin antibody showed the accumulation of insulin in the cells after TCDD treatment (Figure 1D). When the cells were stimulated with 25 mM glucose, the amount of insulin present inside the cells (possibly in the insulin vesicles) was not reduced in the TCDD-treated cells compared with DMSO-treated control cells (Figure 1D). Our results suggest that low-dose TCDD does not affect the insulin synthesis in pancreatic β-cells but only inhibits GSIS, without inducing cell death.

### 2.2. TCDD Reduced Pancreatic Islet Size in Wild Type but Not in AhR-LKO Mice

**Figure 2:**
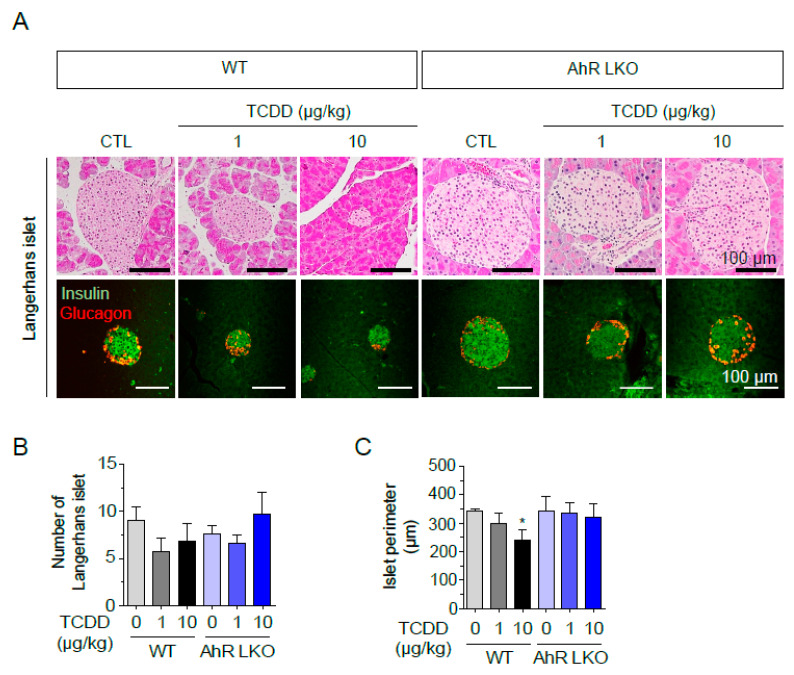
Liver AhR-dependent loss of pancreatic islets by TCDD injection. AhR-LKO mice retain pancreatic islets after TCDD injection. Pancreas was isolated from mice (WT or AhR-LKO) fed a normal chow for 12 weeks after intraperitoneal injection of TCDD (0, 1, or 10 μg/kg). (**A**) H&E staining of paraffin sections of mouse pancreas (top), and double immunostaining of paraffin sections with anti-insulin (green) and anti-glucagon (red) antibodies (bottom). (**B**) Number of Langerhans islets. (**C**) Perimeter per islet calculated by Mingmei camera at magnification: 200× (μm). Data represent the mean ± SEM. * *p* < 0.05 vs. control (*n* = 5~6).

To investigate the effects of low-level exposure to TCDD on the pancreas, wild type (WT) and AhR-LKO mice were injected once with TCDD (0, 1, or 10 μg/kg, *i.p.*), and pancreatic tissues were isolated after 12 weeks on a normal chow. Although the number of islets was not significantly altered by TCDD, H&E-stained paraffin sections of the pancreas showed a dose-dependent reduction in the size, as measured by the perimeter of the islets of Langerhans in WT mice (Figure 2). In contrast, islet size and morphology were not altered in the AhR-LKO mice. Double immunohistochemical staining for glucagon and insulin, representing α- and β-cells, respectively, also showed the smaller islets after TCDD injection in the WT mice. However, in the AhR-LKO mice, the size and number of islets were not affected by the TCDD. The results indicate that TCDD reduces the size of Langerhans islets in the pancreas of WT mice but not in AhR-LKO mice. This suggests that TCDD may induce the secretion of an unknown factor or factors in the liver through AhR activation, resulting in islet death.

### 2.3. Analysis of RNA-Seq Dataset Obtained from TCDD-Treated Mouse Liver

**Figure 3:**
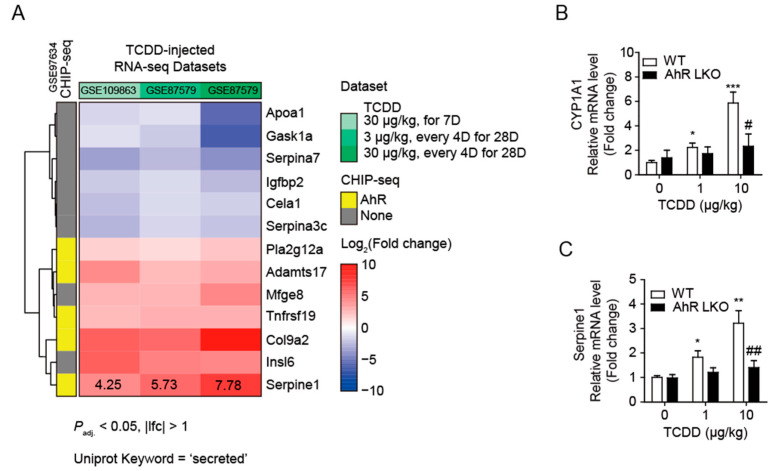
TCDD increased Serpine1 mRNA levels in the liver in an AhR-dependent manner. (**A**) Heatmap of differentially expressed genes, which are annotated with the UniProt keyword “secreted”, in all three RNA-seq datasets. Fold changes (FC) are shown as repressed (blue) or induced (red). (**B**,**C**) Quantitative RT-PCR. Relative mRNA levels of Cyp1A1 (**B**) and Serpine1 (**C**) were determined in mouse liver from WT and AhR-LKO mice *i.p.* injected with TCDD (0, 1, or 10 μg/kg). Mice were on a normal diet, and liver tissues were collected 12 weeks after the TCDD injection. Data represent the mean ± SEM (*n* = 5~7). * *p* < 0.05, ** *p* < 0.01, *** *p* < 0.001 vs. control; ^#^ *p* < 0.05, ^##^ *p* < 0.01 vs. WT.

To understand how the TCDD indirectly affected pancreatic islets, we must first identify the unknown factor(s) secreted by the liver after TCDD injection. To find liver-specific and AhR-dependent secreted proteins, we analyzed liver RNA-seq datasets in the NCBI database (Figure 3). Dataset GSE109863 contains liver gene expression data from mice one week after oral administration of 30 μg/kg TCDD (single exposure). GSE87519 contains data from the livers of mice orally administered various doses (0.01~30 μg/kg) of TCDD and sesame oil vehicle every 4 days for 28 days (total of 7 exposures). Two 3 μg/kg and 30 μg/kg dose data from GSE87519 [17] and GSE109863 were used for transcriptome analysis. In all three groups, 76 and 27 genes were commonly up- or down-regulated (*P*_adj._ < 0.05 and |log_2_FC| > 1), respectively. Additionally, gene ontology analysis revealed that 13 of the 103 differentially expressed genes encode secretory proteins, including *Serpine1* (PAI-1). Figure 3A shows a heatmap of these 13 proteins, and Supplementary Table S1 provides detailed information. The CHIP-seq dataset (GSE97634) clearly shows the genes where the AhR binds to the promoter regions (yellow, Figure 3A). TCDD induced the expression of the AhR-dependent secretory protein *Serpine1* (PAI-1) up to 7.8-fold. To validate the transcriptome analysis results, real-time RT-qPCR was performed using liver samples from the WT and AhR-LKO mice. This confirmed that, as expected, *Serpine1* mRNA increased in an AhR-dependent manner, similar to the positive control Cyp1A1 (Figure 3B,C).

### 2.4. Low-Dose TCDD Increased PAI-1 Expression in Hepa1c1c7 Cells

**Figure 4:**
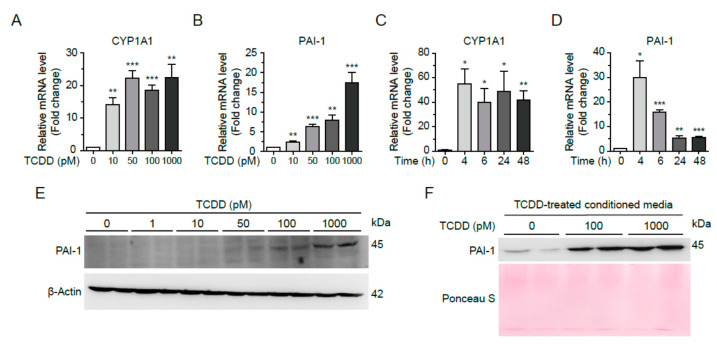
TCDD induced expression and secretion of PAI-1 in Hepa1c1c7 cells. (**A**,**B**) Dose-dependent changes of mRNA. Hepa1c1c7 cells were incubated with TCDD 0, 10, 50, 100, or 1000 pM for 24 h, and mRNA levels of CYP1A1 (**A**) and PAI-1 (**B**) were analyzed by qRT-PCR. (**C**,**D**) Time-dependent changes of mRNA. Hepa1c1c7 cells were incubated with 100 pM TCDD for 0, 4, 6, 24, or 48 h, and mRNA levels of CYP1A1 (**C**) and PAI-1 (**D**) were analyzed by qRT-PCR. The mRNA level was normalized by 18S rRNA. Data represent the mean ± SEM (n = 3). * *p* < 0.05, ** *p* < 0.01, *** *p* < 0.001 vs. DMSO-treated cells. (**E**) Hepa1c1c7 cells were incubated with TCDD for 24 h as designated, and the cell lysates were analyzed by Western blot using an antibody against PAI-1. β-Actin was used as a loading control. (**F**) Cells were incubated with DMSO or TCDD (100 or 1000 pM) in MEMα media containing 10% CS-FBS for 24 h. The culture media were changed to serum-free RPMI 1640, and the cells were incubated for 24 h. Proteins in the collected conditioned media were precipitated by MeOH/CHCl_3_ and analyzed by Western blot using anti-PAI-1 antibody. The Ponceau S-stained image was used as a loading control.

TCDD has been reported to induce PAI-1 gene expression through AhR- and ARNT-dependent mechanisms in mouse hepatoma cell lines [18]. To examine whether TCDD induces PAI-1 expression in liver cells, we incubated Hepa1c1c7 cells with 0, 10, 50, 100, or 1000 pM TCDD for 24 h and then quantified PAI-1 mRNA levels by real-time RT-qPCR. The mRNA level of PAI-1 was increased in a TCDD dose-dependent manner along with CYP1A1 mRNA, a positive control for TCDD response (Figure 4A,B). Monitoring mRNA levels at different time points revealed that 100 pM TCDD treatment resulted in a saturable increase in CYP1A1 mRNA levels at 4 h, whereas the mRNA levels of PAI-1 changed with the duration of the TCDD treatment. The mRNA levels of PAI-1 peaked at 4 h and then decreased at 48 h (Figure 4C,D). The intracellular protein level of PAI-1 was enhanced with increasing TCDD concentration, as seen in the Western blot (Figure 4E). When proteins in conditioned media (CM) were precipitated and analyzed by Western blot, PAI-1 protein in the culture media was also increased by TCDD (100 or 1000 pM) treatment (Figure 4F). Therefore, the liver being exposed to TCDD increases the expression of PAI-1, which is secreted into the blood and affects other organs, including the pancreas. 

### 2.5. TCDD Increased PAI-1 Expression and Secretion in Hepa1c1c7 Cells in an AhR-Dependent Manner

**Figure 5:**
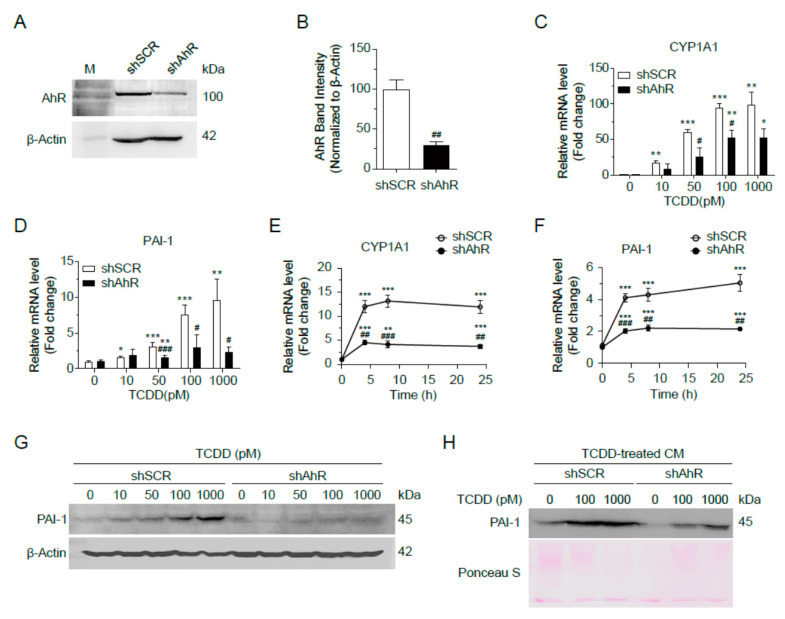
AhR-dependent induction of PAI-1 by TCDD. (**A**,**B**) Hepa1c1c7 cells were transfected with shSCR or shAhR. The expression of AhR was analyzed by Western blot (**A**) and intensities of AhR bands normalized by β-Actin (**B**). (**C**,**D**) Dose-dependent changes of mRNA. shSCR- or shAhR-transfected Hepa1c1c7 cells were incubated with TCDD as indicated for 24 h, and mRNA levels of CYP1A1 (**C**) and PAI-1 (**D**) were analyzed by qRT-PCR. (**E**,**F**) Time-dependent changes of mRNA. shSCR- or shAhR-transfected Hepa1c1c7 cells were incubated with 100 pM TCDD for 0, 4, 6, 24, or 48 h, and mRNA levels of CYP1A1 (**E**) and PAI-1 (**F**) were analyzed by qRT-PCR. The mRNA levels were normalized by 18S rRNA. Data represent the mean ± SEM (*n* = 3). * *p* < 0.05, ** *p* < 0.01, *** *p* < 0.001 vs. DMSO-treated cells. ^#^ *p* < 0.05, ^##^ *p* < 0.01, ^###^ *p* < 0.001 vs. shSCR. (**G**) shSCR- or shAhR-transfected Hepa1c1c7 cells were incubated with TCDD at the indicated concentrations for 24 h and analyzed by Western blot using anti-PAI-1 antibody. β-Actin was used as a loading control. (**H**) shSCR- or shAhR-transfected Hepa1c1c7 cells were incubated with DMSO or TCDD (100 or 1000 pM) in MEMα containing 10% CS-FBS for 24 h, followed by further incubation in serum-free RPMI 1640 for 24 h to collect conditioned media. Proteins in the conditioned media were precipitated by MeOH/CHCl_3_ and analyzed by Western blot using anti-PAI-1 antibody. The Ponceau S-stained image was used as a loading control.

Next, to validate whether TCDD-induced PAI-1 expression is AhR-dependent, we transfected Hepa1c1c7 cells with plasmids containing short hairpin RNA (shAhR or shSCR, control). A Western blot confirmed that AhR was knocked down in the shAhR-transfected cells (Figure 5A,B). When these cells were incubated with 0, 10, 50, 100, or 1000 pM of TCDD for 24 h, mRNA levels of Serpine1 and CYP1A1 increased in a dose-dependent manner in the shSCR-cells, whereas PAI-1 mRNA levels did not increase with increasing TCDD concentrations in the shAhR-cells (Figure 5C,D). Similarly, no time-dependent changes of Serpine1 and CYP1A1 mRNA levels were observed at 100 pM TCDD in the shAhR-cells (Figure 5E,F). Western blots of cell lysates also showed an increase in PAI-1 protein levels in the shSCR-cells but not in the shAhR-cells (Figure 5G). We also observed a decrease in the PAI-1 secreted into the culture media in the shAhR-cells treated with TCDD compared to the shSCR-cells (Figure 5H). These results demonstrated that TCDD induces PAI-1 expression and secretion into the culture media in an AhR-dependent manner.

### 2.6. PAI-1 Secreted from Hepa1c1c7 Cells Induced Apoptosis in RIN-m5F Cells

**Figure 6:**
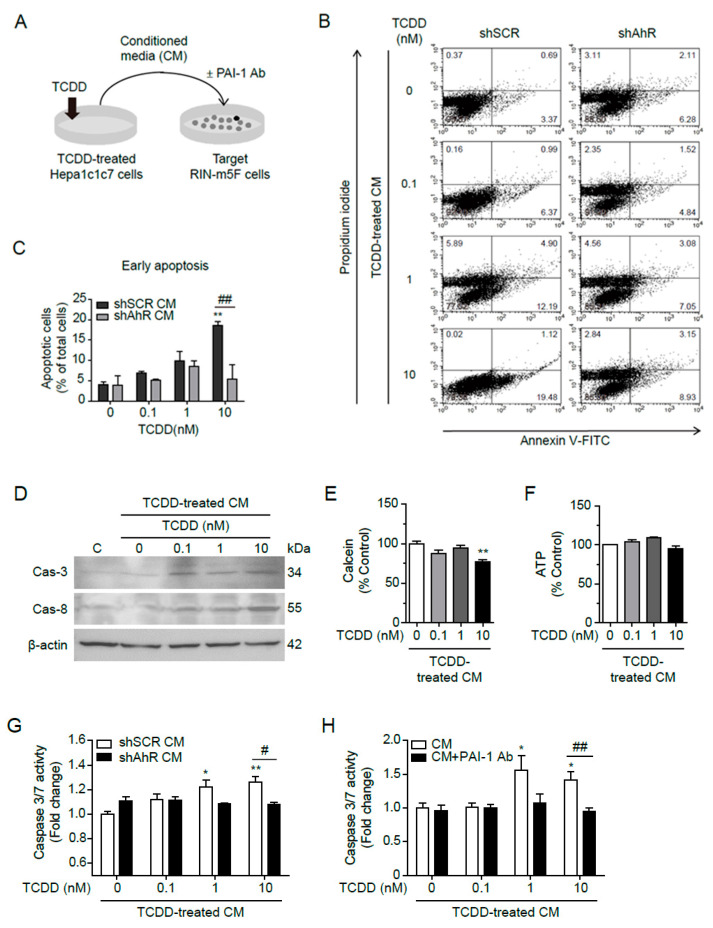
PAI-1 secreted by TCDD from Hepa1c1c7 cells induced apoptosis in RIN-m5F cells. (**A**) Graphical scheme of the conditioned media transfer experiment. Hepa1c1c7 cells were incubated with DMSO or 100 pM TCDD in MEMα containing 10% CS-FBS for 24 h. Conditioned media were collected 24 h after media change to serum-free RPMI 1640, and RIN-m5F cells were incubated in the conditioned media containing 10% CS-FBS for 24 h, with or without PAI-1 antibody. (**B**) After incubation with the conditioned media from shSCR- or shAhR-transfected Hepa1c1c7 cells, the RIN-m5F cells were stained with Annexin V and propidium iodide (PI), and apoptosis was monitored through flow cytometry. The X and Y axes show Annexin V and PI staining markers, for apoptosis and necrosis, respectively. Each plot is divided into four regions. The lower-left region shows cells negative for both Annexin V and PI (living cells); the lower-right region shows cells positive for Annexin V only (early apoptotic cells); the upper-left region shows cells positive for PI only (necrotic cells); the upper-right region shows cells positive for both. (**C**) Quantitative graph of early apoptotic cells as % of total cells. (**D**) Western blot against Caspase-3 and Caspase-8 after incubation with conditioned media for 24 h (C: control for incubation with conditioned media). β-Actin was used as a loading control. (**E**,**F**) After incubation in conditioned media, the cell viability and mitochondrial activity were measured by calcein (**E**) and intracellular ATP contents (**F**), respectively. (**G**,**H**) A Caspase-3/7 assay was performed after incubation with conditioned media from shSCR- or shAhR-transfected cells (**G**) or from Hepa1c1c7 cells with or without PAI-1 antibody (**H**). Data represent the mean ± SEM (*n* = 3~5). * *p* < 0.05, ** *p* < 0.01 vs. control; ^#^ *p* < 0.05, ^##^ *p* < 0.01 vs. shSCR.

To investigate whether PAI-1 secreted from the liver leads to β-cell failure in T2D patients, the effects of PAI-1 on pancreatic β-cell apoptosis were examined. Conditioned medium (CM) was collected from Hepa1c1c7 cells after TCDD treatment, and RIN-m5F cells were incubated with TCDD-treated CM containing 10% CS-FBS for 24 h (Figure 6A). The apoptosis of RIN-m5F cells induced by the TCDD-treated CM was analyzed by flow cytometry. The RIN-m5F cells incubated with TCDD-treated CM for 24 h were stained with Annexin V and PI. The number of early apoptotic cells were increased after incubation with CM from 10 nM TCDD-treated shSCR cells (Annexin V-positive, PI-negative) (Figure 6B,C). 

A Western blot of the RIN-m5F cell lysates demonstrated that the TCDD-treated CM from Hepa1c1c7 cells increased Caspase-3 and Caspase-8 protein levels dose-dependently, although TCDD was not directly treated in the RIN-m5F cells (Figure 6D). Calcein activity, a marker for cell viability, showed a 22.8% reduction after incubation with CM from 10 nM TCDD-treated Hepa1c1c7 cells (Figure 6E), but intracellular ATP content, a marker for mitochondrial activity, was not altered (Figure 6F). When Caspase-3/7 activity was determined, CM from shSCR-transfected cells incubated with 1 or 10 nM TCDD increased Caspase-3/7 activity in the RIN-m5F cells, while CM from shAhR-transfected cells had no effect on Caspase-3/7 activity in the RIN-m5F cells (Figure 6G). Furthermore, to confirm whether PAI-1 indeed induced Caspase-3/7-dependent apoptosis of the RIN-m5F cells, PAI-1 in TCDD-treated CM was depleted by incubation with an anti-PAI-1 antibody. The CM treated with PAI-1 antibody, i.e., PAI-1-depleted CM, failed to activate Caspase-3/7 activity (Figure 6H). The results clearly showed that PAI-1 secreted from Hepa1c1c7 cells induced Caspase-3/7-dependent apoptosis in RIN-m5F cells, suggesting that circulating PAI-1 may impair β-cell function.

## 3. Discussion

In this study, we demonstrated that TCDD, a representative EPC and AhR ligand, increased intracellular insulin levels but inhibited GSIS in RIN-m5F pancreatic β-cells. Additionally, mice injected with a single low dose of TCDD exhibited a reduction in the size of pancreatic islets. Our focus on low-dose TCDD (100 pM) effects uniquely highlights that low-dose TCDD stimulates PAI-1 expression and secretion in Hepa1c1c7 cells through an AhR-dependent mechanism, leading to an early stage of apoptosis in RIN-m5F cells. Unlike other studies that examined the direct effects of TCDD on β-cells without involving the secretory factor PAI-1 [19,20], our findings reveal a hepatocyte-mediated pathway. Since both AhR ligands and PAI-1 are elevated in the blood of T2D patients, this study suggests a novel mechanistic link between AhR ligand-induced PAI-1 production and pancreatic β-cell failure in T2D.

T2D is a chronic, heterogeneous syndrome characterized by insulin resistance and pancreatic β-cell dysfunction or death [7,21]. EPCs can disrupt energy homeostasis by affecting multiple cellular processes involved in energy production and utilization [22,23]. However, the specific mechanisms by which EPCs induce insulin resistance or pancreatic β-cell dysfunction, and the causative factor behind pancreatic β-cell failure, remain unclear. Our in vivo TCDD-injected mouse model showed that a single injection of low-concentration TCDD clearly reduced the mass of pancreatic islets in WT mice but not in AhR-LKO mice. This indicates that TCDD does not directly destroy pancreatic islets but rather involves liver-derived secretory proteins in β-cell failure. This finding provides a critical clue: the unidentified factor is a liver-derived secretory protein, its expression is increased by TCDD in an AhR-dependent manner, and it is elevated in the blood of T2D patients. Through transcriptome analysis, we identified *Serpine1* (Serpin family E member 1) as one of the candidate genes for this unidentified factor.

*Serpine1* encodes PAI-1, a fast-acting serine protease inhibitor that regulates the fibrinolytic system by inhibiting tissue plasminogen activator (tPA) and urokinase-type plasminogen activator (uPA) [14]. Thus, PAI-1 is responsible for the controlled degradation of blood clots. PAI-1 is commonly associated with cardiovascular disease (CVD) or T2D, reflecting the global prothrombotic and inflammatory milieu of insulin resistance [24,25]. Moreover, it has been shown that PAI-1 is more than an innocent bystander in the pathogenesis of ischemic heart disease [26,27]. Elevated levels of PAI-1 can increase the risk of atherosclerosis and accelerate the progression of vascular disease [28,29]. There is also a strong positive relationship between PAI-1 and obesity, diabetes, and metabolic syndrome, making it central to the pathophysiological process [30]. Circulating PAI-1 is elevated in patients with T2D, but this is not necessarily true for patients with T1D [16], suggesting that the primary cause of elevated PAI-1 levels in T2D may be related to obesity and insulin resistance rather than elevated glucose levels. 

Insulin, triglycerides, and fatty acids are among the factors that have been shown to increase PAI-1 expression. So far, TCDD is known to directly induce *Serpine1* through an AhR- and ARNT-dependent mechanism in mouse liver cancer cell lines [18,31]. AhR is a multifunctional nuclear receptor that mediates the toxic response induced by POPs and plays a pivotal role in the detoxification of xenobiotics [1,32,33]. In addition, the liver produces many endogenous AhR ligands, positioning AhR as a novel therapeutic target for liver diseases [18,34]. Interestingly, CHIP-seq analysis revealed that AhR binds to the promoter region of PAI-1, indicating that EPC AhR ligands may be among the factors that increase PAI-1 expression. However, while PAI-1 induction by AhR ligands has been reported [18,31], the association between EPC AhR ligands and T2D, obesity, and metabolic syndrome is not clearly understood. In our animal and cellular models, we confirmed that TCDD administration to mice elevated the expression of PAI-1 in the liver and its secretion into the blood in an AhR-dependent manner.

Elevated PAI-1 levels in patients with T2D, obesity, and metabolic syndrome are also associated with low-grade chronic inflammation. A high-fat diet increased PAI-1 expression and the number of macrophages in visceral white adipose tissue, whereas genetic or pharmacological inhibition of PAI-1 reduced macrophage infiltration [27]. However, the effects of exogenous PAI-1 on cells, particularly on pancreatic β-cells, have not been well studied [28]. PAI-1 interacts with tPA or uPA to inhibit fibrinolysis, and both PAI-1-tPA/uPA complexes and free PAI-1 can bind to low-density lipoprotein receptor-related protein 1 (LRP1) [35,36]. PAI-1 treatment induces internalization of the PAI-1-(tPA/uPA)-LRP1 complex by endocytosis, leading to PAI-1 degradation. LRP1, a scavenger receptor mediating various signaling pathways, is a key modulator of β-cell function in T2D [37]. In β-cells, LRP1 maintains PPARγ2 levels at manageable concentrations and prevents Erk activation by glucose and Ca^2+^ [38]. Other studies have reported that the interaction between PAI-1 and LRP1 mediates cell migration and proliferation [39,40,41,42]. Indeed, failure of compensatory proliferation of β-cells leads to hyperglycemia and insulin dependence in T2D patients, and ablation of LRP1 in β-cells from mice fed a high fat-diet impairs β-cell function and proliferation [38]. Therefore, TCDD-mediated increased circulating PAI-1 may bind to LRP1, resulting in degradation and/or down-regulation of LRP1 and, consequently, β-cell insufficiency. More importantly, circulating PAI-1 can induce β-cell apoptosis (Figure 6), contributing to the relative β-cell deficiency in T2D patients. Although the involvement of LRP1 in β-cell apoptosis was not tested in this study, the use of PAI-1 antibody clearly attenuated apoptosis induced by the conditioned medium of TCDD-treated Hepa1c1c7 cells (Figure 6H). 

PAI-1-induced apoptosis of RIN-m5F cells was Caspase-3/7-dependent, triggering by cytochrome c release from mitochondria. Although a decrease in ATP content was not observed in this study, mitochondrial dysfunction may initiate the release of cytochrome c from mitochondria, leading to apoptosis. These results are in good agreement with the hypothesis that exposure to AhR ligands or TCDD is an important risk factor for β-cell failure and T2D [43,44]. Other studies have shown that cells incubated with diabetic serum containing high levels of AhR ligands inhibited mitochondrial function, mitochondrial oxygen consumption rate, and intracellular ATP contents, or increased ROS and mitochondrial fragmentation [5]. Therefore, PAI-1 in diabetic serum may act as a mitochondrial inhibitor.

In conclusion, this study demonstrates that β-cell failure seen in the late stages of T2D can be caused by chronic exposure to EPCs which are AhR ligands. EPCs increase the secretion of PAI-1 from the liver in an AhR-dependent manner, and the secreted PAI-1 circulates in the body, inducing β-cell apoptosis and exacerbating T2D. Our findings provide a novel molecular mechanism for how EPCs contribute to the loss of functional β-cell mass in diabetes development. The EPC-PAI-1 axis may be a major contributor to pancreatic β-cell failure in T2D. Further research is needed to determine whether diabetes complications and other PAI-1-related diseases, such as arteriosclerosis and stroke, are also caused by AhR ligand exposure. 

## 4. Materials and Methods

### 4.1. Cell Culture and Conditioned Media

Hepa1c1c7 mouse liver cells (ATCC^®^ CRL-2026™, Manassas, VA, USA) were cultured in α-modified Eagle’s Minimum Essential Medium (MEMα), and RIN-m5F rat pancreatic β-cells (ATCC^®^ CRL-2058™) were cultured in Roswell Park Memorial Institute (RPMI) 1640 medium. Both media were supplemented with 10% fetal bovine serum (FBS), 100 U/mL penicillin, and 100 μg/mL streptomycin and maintained at 37 °C in a humidified incubator with an atmosphere of 95% O_2_ and 5% CO_2_. The Hepa1c1c7 cells were incubated for 16 h in serum-deficient medium containing 0.5% charcoal-stripped FBS (CS-FBS) and treated with 100 pM TCDD for 24 h. The TCDD-treated cells were harvested for subsequent analysis via Western blot and real-time RT-qPCR.

For the collection of conditioned media from the Hepa1c1c7 cells, cells (4 × 10^4^ cells/well in a 96-well plate) were treated with TCDD (0, 0.1, 1, 10 nM) for 24 h in MEMα containing 10% CS-FBS, washed with serum-free RPMI 1640, and incubated for another 24 h in serum-free RPMI 1640. The culture media were then collected and centrifuged at 1000× *g* for 10 min at 4 °C to remove cell debris. The supernatant was used as the conditioned medium for subsequent experiments. 

To deplete PAI-1, the conditioned medium was placed in an Eppendorf tube and incubated with anti-PAI-1 antibody (1:30, RabMab, EPR21850-82, Abcam, Cambridge, UK) for 2 h at room temperature with shaking. Conditioned media with or without PAI-1 depletion were supplemented with 10% FBS and used to culture RIN-m5F cells for 24 h for further analysis.

### 4.2. Animal and Experimental Design

Ahr flox (Ahr^tm3.1Bra^/J) mice and C57BL/6J (wild type, WT) mice were purchased from the Jackson Laboratory (Bar Harbor, ME, USA). To obtain liver-specific AhR knockout mice (AhR-LKO), mice harboring a floxed allele of Ahr flox were crossed with transgenic albumin-Cre mice (C57BL/6J-TgN (Alb-cre) Gto/J, a generous gift from Dr. GT Oh of Ewha Womans University, Seoul, Republic of Korea). Male C57/BL6 (WT) and AhR-LKO mice were divided into six groups (*n* = 5/group): a corn oil-injected control (CTL) and five TCDD-injected groups (0.5, 1, 2, 5, or 10 μg/kg). The mice were maintained at room temperature (22–24 °C), 50–60% humidity, with a 12 h light/dark cycle in a specific pathogen-free barrier facility with free access to food and water. The mice were injected intraperitoneally with TCDD (0.5, 1, 2, 5, or 10 μg/kg) or corn oil and maintained on a normal diet for 12 weeks. The animals were maintained and treated in accordance with the Principles of Laboratory Animal Care (NIH Publication No. 85-23, revised 1985) and the Animal Care and Use Guidelines (KHSASP-20-163) of Kyung Hee University, Seoul, Republic of Korea.

### 4.3. Paraffin Block Preparation

Mice were anesthetized by *i.p.* injection of chloral hydrate (40 mg/kg), transcardially perfused with a saline solution containing 0.5% sodium nitrate and heparin (10 U/mL), and then fixed with 4% paraformaldehyde dissolved in 0.1 M phosphate buffer (PB, pH 7.2). Mouse liver, white adipose tissue (WAT), brown adipose tissue (BAT), and pancreas tissues were postfixed with 4% paraformaldehyde in 0.1 M PB at 4 °C overnight. The fixed tissues were trimmed into appropriate size and shape and placed in embedding cassettes. The paraffin embedding schedule was as follows (17 h total): 70% ethanol, two changes, 1 h each; 80% ethanol, one change, 1 h; 95% ethanol, one change, 1 h; 100% ethanol, three changes, 1.5 h each; xylene, three changes, 1.5 h each; paraffin wax (58–60 °C), two changes, 2 h each; embedding tissues in paraffin blocks. Pancreas sections (5 μm thick) were incubated with rabbit anti-insulin antibody (Cell Signaling Technology, Danvers, MA, USA) for pancreatic β-cells and mouse anti-glucagon antibody (Abcam, Cambridge, UK) for α-cells. Alexa 488-anti-rabbit and Alexa 555-anti-mouse (Invitrogen, Carlsbad, CA, USA) were used as secondary antibodies for the β-cells and α-cells, respectively.

### 4.4. Hematoxylin and Eosin (H&E) Staining

The paraffin blocks were cut into 5 µm thick slices and mounted on SuperFrost microscope slides (VWR, Radnor, PA, USA). After step-wise deparaffinization and rehydration, the slides were stained with hematoxylin and eosin (Abcam) according to standard protocols.

### 4.5. ELISA and Immunocytochemistry

For analysis of glucose-stimulated insulin secretion, RIN-m5F cells were cultured in 6-well plates for ELISA or in confocal dishes for immunocytochemistry. The cells were incubated for 24 h in RPMI 1640 medium containing 10% CS-FBS and were incubated with 100 pM TCDD for 24 h. After TCDD treatment, the cells were washed with Hank’s balanced salt solution (HBSS: 20 mM HEPES pH 7.4, 145 mM NaCl, 5 mM KCl, 1 mM MgCl_2_, 2 mM CaCl_2_, 5 mM glucose) and then incubated with 5 mM or 25 mM glucose for 1 h in HBSS to induce insulin secretion. The insulin concentration in the supernatants (100 μL) was determined using a rat insulin ELISA kit (Thermo Fisher Scientific, Waltham, MA, USA) and was normalized to the protein concentration of the cells used.

After glucose stimulation on confocal dishes, the cells were washed with phosphate-buffered saline (PBS) and were fixed with 4% PFA. The cells were permeabilized with PBS containing 0.1% Triton X-100 for 10 min and were incubated with PBS containing 1% BSA for 30 min. Immunocytochemistry (ICC) was performed with an anti-insulin antibody (1:500, Santa Cruz, Dallas, TX, USA). Nuclei were stained with 1 μg/mL Hoechst 33342 (Molecular Probes, Eugene, OR, USA) for 20 min in PBS at room temperature. Cell images were captured using an LSM700 laser-scanning confocal microscope (Carl Zeiss, Oberkochen, Germany).

### 4.6. Western Blot Analysis

Harvested cell lysates were separated by 10% or 15% SDS-PAGE and analyzed by Western blot [45]. Primary antibodies against AhR (1:2000, Enzo Life Sciences, Farmingdale, NY, USA), insulin (1:1000, Cell Signaling Technology, Danvers, MA, USA), PAI-1 (1:1000, Abcam, Cambridge, UK), Caspase-3 (1:1000, Santa Cruz, Dallas, TX, USA), and Caspase-8 (1:1000, Santa Cruz, Dallas, TX, USA) were purchased from commercial sources. Anti-mouse and anti-rabbit secondary antibodies were purchased from Cell Signaling Technology. Equivalent protein loading was verified with an anti-β-actin antibody (Santa Cruz) or Ponceau S staining of nylon membrane. 

### 4.7. Quantitative Real-Time RT-PCR (RT-qPCR)

Total RNA was isolated from cultured cells using TRIzol™ reagent (Invitrogen), reverse-transcribed using 10 ng random hexamers (Invitrogen) and MMLV reverse transcriptase (Promega Corporation, Madison, WI, USA), and then subjected to SYBR Green dye-based quantitative real-time PCR as previously described [45]. The general PCR conditions were 5 min at 95 °C, 40 cycles of 5 s at 95 °C, and 30 s at 60 °C. The PCR reaction was performed on a Roter-Gene Q (Qiagen, Hilden, Germany). The amount of mRNA was normalized by simultaneous measurements of 18S rRNA. The relative gene expression level was determined by the 2^−ΔΔCt^ method. Primer sequences for RT-qPCR were as follows: Serpine1 (5′-TCTCTTTGTGGTTCGGCACA-3′, 5′-TTCGTCCCAAATGAAGGCGT-3′), Cyp1A1 (5′-TCCGGCATTCATCCTTCGTC-3′, 5′-ACAGTTCCCGGTCATGGTTA-3′), and 18S rRNA (5′-GAGCGAAAGCATTTGCCAAG-3′, 5′-GGCATCGTTTATGGTCGGAA-3′).

### 4.8. Cell Viability Assay

Cell viability was measured using Calcein AM (Molecular Probes, Eugene, OR, USA). RIN-m5F cells (6 × 10^4^ cells/well) were cultured on black 96-well culture plates in RPMI 1640 with 10% FBS for 24 h. The media were removed and replaced with conditioned media collected from Hepa1c1c7 cells with 10% CS-FBS. After 24 h, 0.5 μM Calcein AM was added to the cells without changing the media and incubated at 37 °C for 1 h. The media were replaced with 100 μL DPBS and the fluorescence intensity was measured using a fluorescence plate reader (SpectraMax^®^ Gemini™ EM, Molecular Devices, Sunnyvale, CA, USA) at 485 nm (excitation) and 535 nm (emission).

### 4.9. Luciferase-Based Intracellular ATP Assay

Intracellular ATP contents were measured by luciferin–luciferase reaction using the CellTiter-Glo^®^ luciferase kit (Promega) according to the manufacturer’s instructions. Briefly, 50 μL of cell lysates were mixed with 50 μL of luciferin–luciferase reaction buffer and incubated at 20 °C for 10 min. The luminescence signal was measured using a Centro LB 960 luminometer (Berthold, Bad Wildbad, Germany). A background luminescence value in control wells containing medium without cells was subtracted from the signal. The ATP levels were normalized to the protein concentration. All data were expressed as percentages of control after calculation. 

### 4.10. Apoptosis Assay

The Caspase-Glo^®^ 3/7 assay (Promega) was used to assess the Caspase-3 and -7 activity after treatment. Briefly, RIN-m5F cells (6 × 10^4^ cells/well) were seeded in 96-well plates and incubated for 24 h in conditioned media from Hepa1c1c7 cells containing 10% CS-FBS at 37 °C. The Caspase-Glo^®^ 3/7 assay was performed according to the manufacturer’s instructions. The luminescence intensity was then measured using a Centro LB 960 luminometer (Berthold).

### 4.11. Transfection

Transfection was performed using Lipofectamine™ 2000 Transfection Reagent (Invitrogen) according to the manufacturer’s instructions [45]. For AhR knock-down in Hepa1c1c7 cells, double-stranded DNA oligonucleotides for short hairpin RNA against mouse AhR (shAhR) containing the BamHI-shAhR (sense)-XhoI loop-antisense with T5-EcoRI sequences (5′-gatccg actctctgttcttaggctc ttctcgaga gagcctaagaacagagagt tttttggaag-3′) were cloned into the RNAi-Ready pSIREN-RetroQ vector (Clontech, Mountain View, CA, USA). A pSIREN-RetroQ-shSCR (shSCR) containing a scrambled control shRNA (5′-gatccg caacaagaagacgcgaatc ttctcgaga gattcgcgtcttcttgttg tttttggaag-3′) was also generated. Sequence verification was performed by DNA sequencing and NCBI blast searches. pSIREN-RetroQ-shAhR (shAhR) or shSCR plasmids were transfected into 70% confluent Hepa1c1c7 cells in 6-well plates. Stably transfected colonies were selected using 1 μg/mL puromycin for 4 weeks.

### 4.12. Analysis of Protein in Conditioned Media

All experiments were performed at room temperature. After TCDD treatment, culture media from Hepa1c1c7 cells were centrifuged at 1000× *g* for 10 min at 4 °C to remove cell debris present in the conditioned media samples. To precipitate proteins in the conditioned media, the conditioned media were mixed with methanol (MeOH) and chloroform (CHCl_3_) in a ratio of 500:500:125 (media:MeOH:CHCl_3_). After incubation for 10 min at RT, the samples were centrifuged at 16,000× *g* for 10 min at 4 °C. Since the proteins remained at the phase boundary between the aqueous MeOH phase and the CHCl_3_ phase, the aqueous phase was removed from the top of the sample, and the protein precipitates were washed with 1 mL MeOH. After centrifugation at 16,000× *g* for 10 min at 4 °C, the protein pellets were dried and resuspended in PRO-PREP protein extraction solution (iNtRON Biotechnology, Seongnam, Republic of Korea) for subsequent SDS-PAGE analysis.

### 4.13. Annexin V Staining

Cellular apoptosis was determined using the eBioscience™ Annexin V Apoptosis Detection Kit FITC (Thermo Fisher Scientific, Basel, Switzerland) according to the manufacturer’s instructions. In brief, RIN-m5F cells were seeded in 6-well plates at a density of 1 × 10^6^ cells per well and incubated overnight at 37 °C in RPMI 1640 supplemented with 10% FBS for 24 h. The media were replaced with conditioned media obtained from Hepa1c1c7 cells. After 24 h, the RIN-m5F cells were trypsinized, washed with DPBS, and washed again with 1× Annexin-Binding buffer. The cells were then resuspended in 100 μL of 1× Annexin-Binding buffer containing 5 μL of Annexin V-FITC and 2.5 μL of propidium iodide (PI) staining solution, followed by incubation at RT for 15 min. The cells were then washed with 1× Annexin-Binding buffer and analyzed using a MACSQuant Analyzer 10 flow cytometer (Miltenyi Biotec, Bergisch Gladbach, Germany). FlowJo software (v10.6.2, FlowJo LLC, Ashland, OR, USA) was used for data analysis.

### 4.14. Transcriptome Data Analysis

Previously published RNA-seq and CHIP-seq datasets were used in this study [17,46,47]. The RNA-seq datasets (GSE109863 and GSE87519) were obtained from Gene Expression Omnibus. The aligned read counts for each gene were imported into the R environment v4.2.2 and preprocessed using the edgeR package v3.40.0 [48]. Differential expression analysis was performed with the limma package v3.54.0 [49], using an empirical Bayesian method. Differentially expressed genes (DEGs) were identified if they had an adjusted *p*-value (*P*_adj._) of less than 0.05 and a log_2_(Fold change) (Log_2_FC) greater than 1. To investigate the AhR dependency of genes, the ChiP-seq dataset (GSE97634) was integrated. Genes with a false discovery rate of ≤0.05 were considered to have significant AhR binding.

### 4.15. Statistical Analysis

Data are expressed as mean ± standard error of the mean (SEM). Statistical significance between groups was assessed by an unpaired Student’s *t*-test using InStat ver. 8 (GraphPad Software, San Diego, CA, USA). Significance was defined as a *p* value < 0.05.

## 5. Conclusions

This study demonstrates that β-cell failure seen in the late stages of T2D can be caused by chronic exposure to AhR ligands. EPCs increase the secretion of PAI-1 from the liver in an AhR-dependent manner, and the secreted PAI-1 circulates in the body, inducing β-cell apoptosis and exacerbating T2D. Our findings provide a novel molecular mechanism for how EPCs contribute to the loss of functional β-cell mass in diabetes development. The EPC-PAI-1 axis may be a major contributor to pancreatic β-cell failure in T2D. Further research is needed to determine whether diabetes complications and other PAI-1-related diseases, such as arteriosclerosis and stroke, are also caused by AhR ligand exposure. 

## Data Availability

The original contributions presented in this study are included in the article/Supplementary Materials. Further inquiries can be directed to the corresponding author(s).

## Supplementary Materials

The supporting information can be downloaded at https://www.mdpi.com/article/10.3390/ijms252211974/s1.
