# Supplementary material for: Dioxin-Induced PAI-1 Expression: A Novel Pathway to Pancreatic β-Cell Failure in Type 2 Diabetes
Source: Int J Mol Sci. 2024 Nov 7;25(22):11974. doi: 10.3390/ijms252211974 (PMC11594116; doi:10.3390/ijms252211974)
Supplement: Supplementary file 1 [file ijms-25-11974-s001.zip › ijms-3285055-supplementary.pdf]

Supplementary Table S1. List of 13 genes annotated as 'secreted'.

ENTREZID	SYMBOL	GENENAME	TCDD_1	TCDD_2	TCDD_3	CHIP-seq
11806	Apoa1	apolipoprotein A-I	-1.55906	-1.17243	-5.49471	None
245050	Gask1a	golgi associated kinase 1A	-1.16598	-1.83759	-6.15324	None
331535	Serpina7	serine (or cysteine) peptidase inhibitor, clade A (alpha-1 antiproteinase, antitrypsin), member 7	-3.54674	-2.46288	-4.04829	None
16008	Igfbp2	insulin-like growth factor binding protein 2	-1.82908	-1.22569	-2.57881	None
109901	Cela1	chymotrypsin-like elastase family, member 1	-2.20739	-1.3432	-1.70639	None
16625	Serpina3c	serine (or cysteine) peptidase inhibitor, clade A, member 3C	-2.61836	-1.44469	-2.13349	None
66350	Pla2g12a	phospholipase A2, group XIIA	1.716383	1.356751	2.06774	AhR
233332	Adamts17	a disintegrin-like and metallopeptidase (reprolysin type) with thrombospondin type 1 motif, 17	4.256015	2.462875	3.036405	AhR
17304	Mfge8	milk fat globule EGF and factor V/VIII domain containing	2.797613	2.653158	4.503211	None
29820	Tnfrsf19	tumor necrosis factor receptor superfamily, member 19	2.590191	2.975945	2.96406	AhR
12840	Col9a2	collagen, type IX, alpha 2	6.053097	5.784023	11.36311	AhR
27356	Insl6	insulin-like 6	6.081238	4.725018	4.548868	None
18787	Serpine1	serine (or cysteine) peptidase inhibitor, clade E, member 1	4.246021	5.729618	7.784394	AhR

These 13 genes were selected among common 103 differentially expressed genes in all three RNA-seq datasets. Fold changes (FC) are shown as repressed (negative) or induced (positive). Analysis of CHIP-seq dataset (GSE97634) identified AhR-dependent genes (AhR).

TCDD1, GSE109863, 30 μg/kg TCDD (single exposure) for 7 days

TCDD2, GSE87519, 3 μg/kg TCDD every 4 days for 28 days (total of 7 exposures)

TCDD3, GSE87519, 30 μg/kg TCDD every 4 days for 28 days (total of 7 exposures)
